# Supplementary material for: Overcoming C60-induced interfacial recombination in inverted perovskite solar cells by electron-transporting carborane
Source: Nat Commun. 2022 Dec 2;13:7454. doi: 10.1038/s41467-022-34203-x (PMC9718752; doi:10.1038/s41467-022-34203-x)
Supplement: Supplementary file 1 — Supplementary Information [file 41467_2022_34203_MOESM1_ESM.pdf]

Supplementary Information

Overcoming C_{60}-induced interfacial recombination in inverted perovskite solar cells by stable electron transporting carborane

Ye et al.

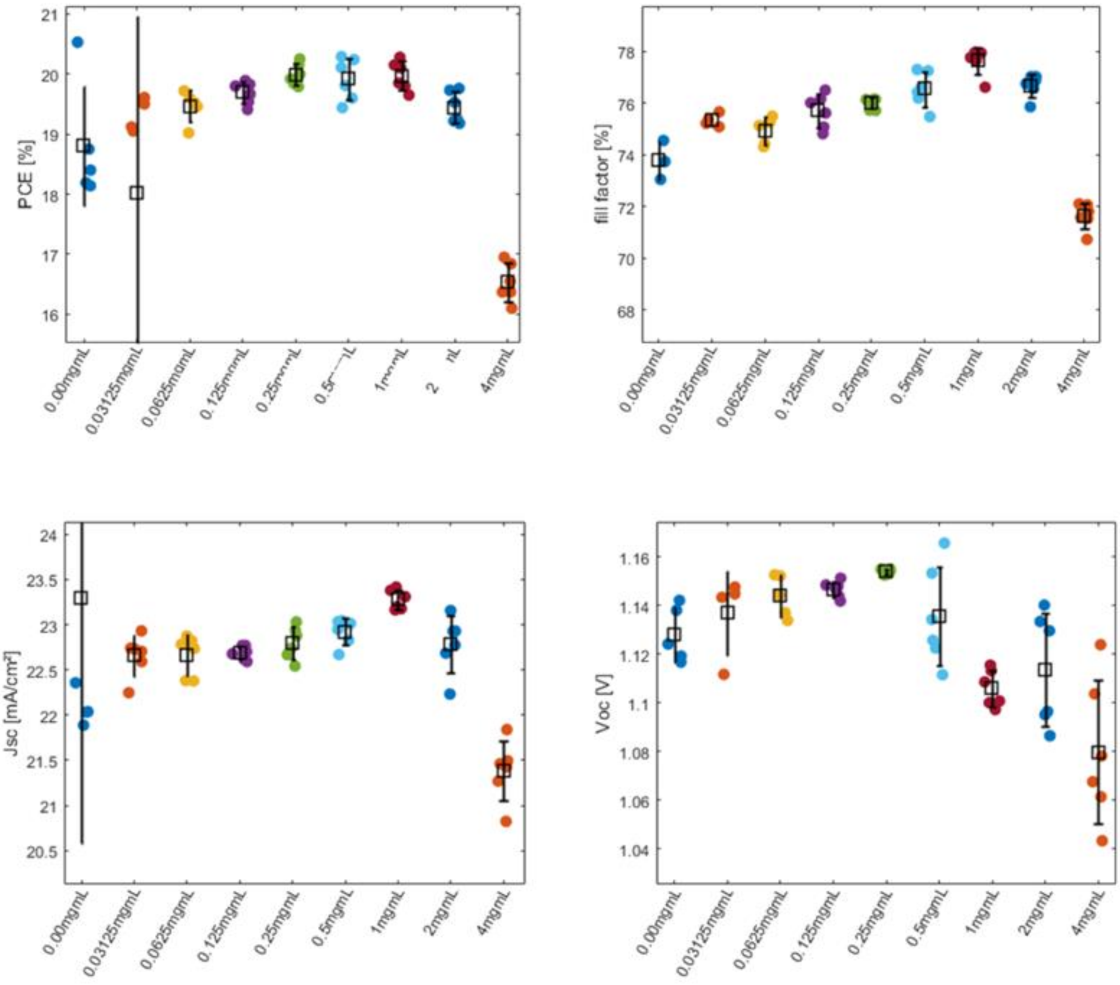

Supplementary Figure 1. Investigation into the effect of PMMA as an interlayer in our triple cation perovskite solar cells where the device structure is ITO/PTAA:PFN/Perovskite/PMMA/C_{60}/BCP/Cu. We have systematically doubled the concentration of the PMMA solution from one point to the next.

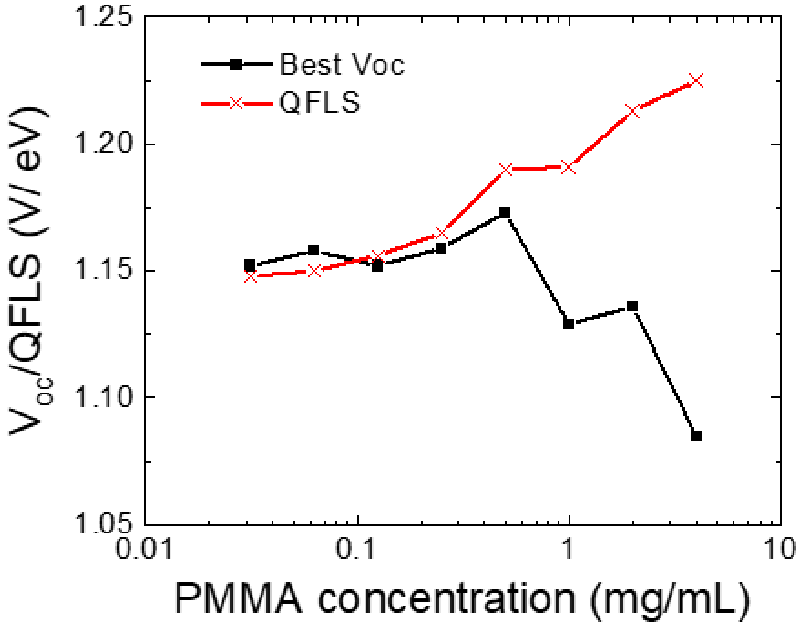

Supplementary Figure 2. QFLS and best V_{OC} of the devices based on different concentration of PMMA. A strong mismatch between the V_{OC} and the QFLS can been attributed to low mobility interlayers[1], and this begins to appear at 1 mg/mL PMMA. This indicates that the continuity/ thickness of the PMMA interlayer at 1 mg/mL is enough to significantly reduce the mobility of the transport layer assembly. In fact, the QFLS continues to increase all the way up to the 4 mg/mL sample which should be due to the film thickness increasing and physically separating the perovskite from the C_{60}. Clearly there is still some electronic contact still at 4 mg/mL as the devices still function further demonstrating the benefit of a lithographic process as the thickness could be increased further still.

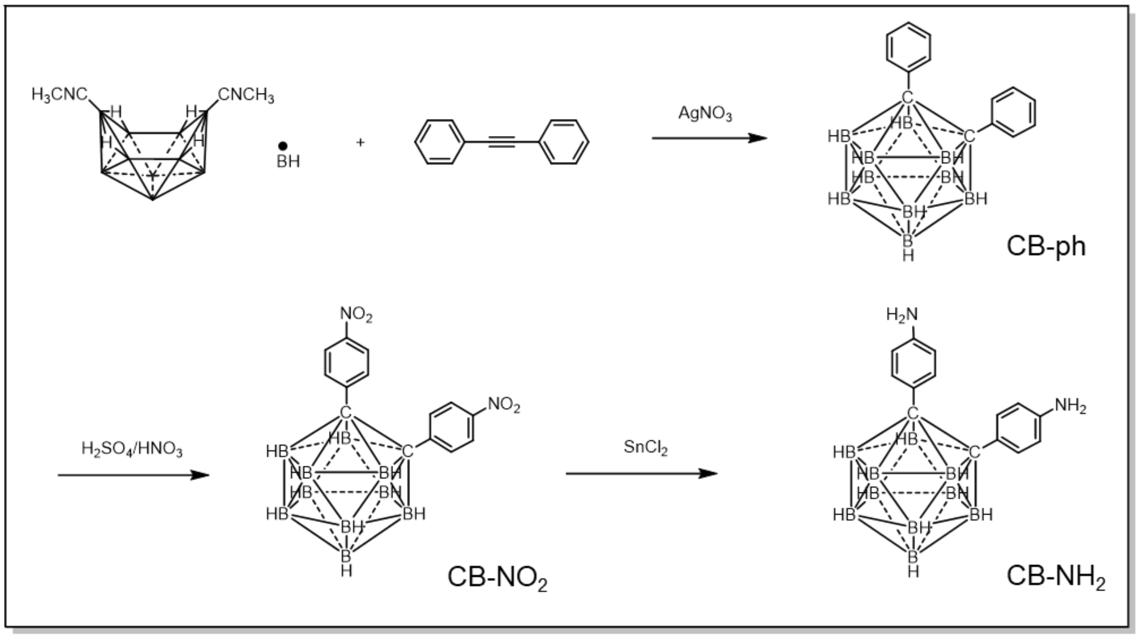

Supplementary Figure 3. Synthetic routes of CB-NH₂.

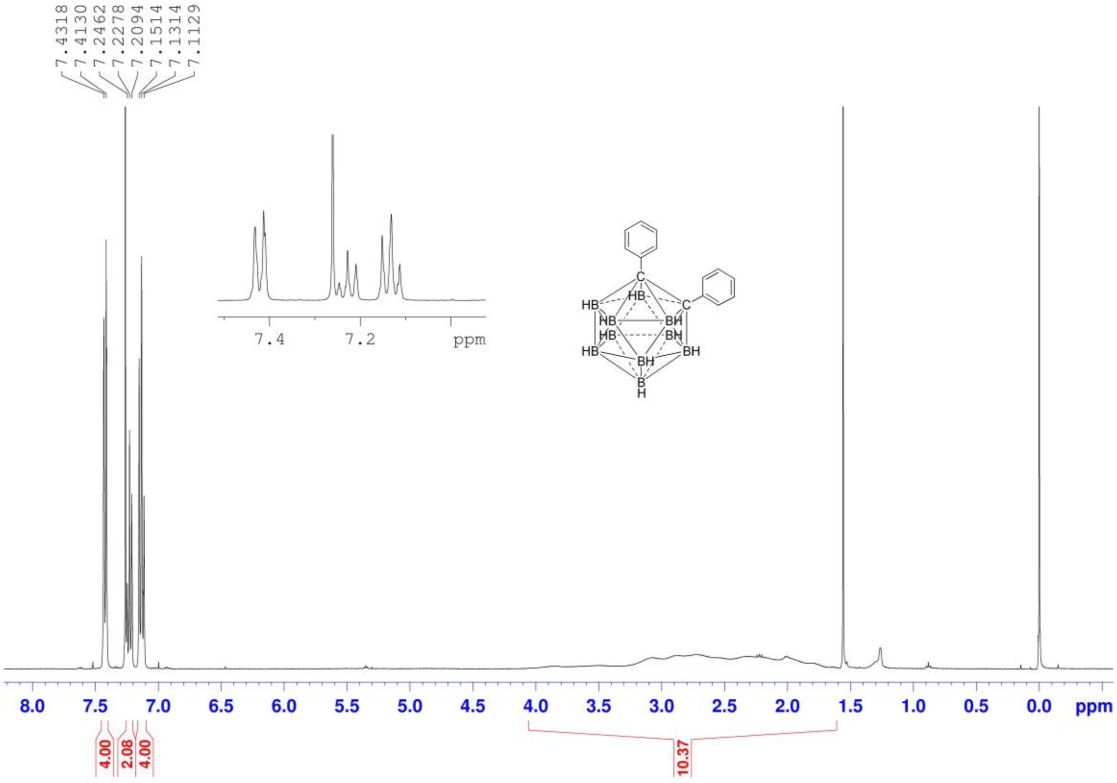

Supplementary Figure 4. ¹H NMR spectrum of compound CB-ph ($CDCl_3$/TMS, 400 MHz, ppm).

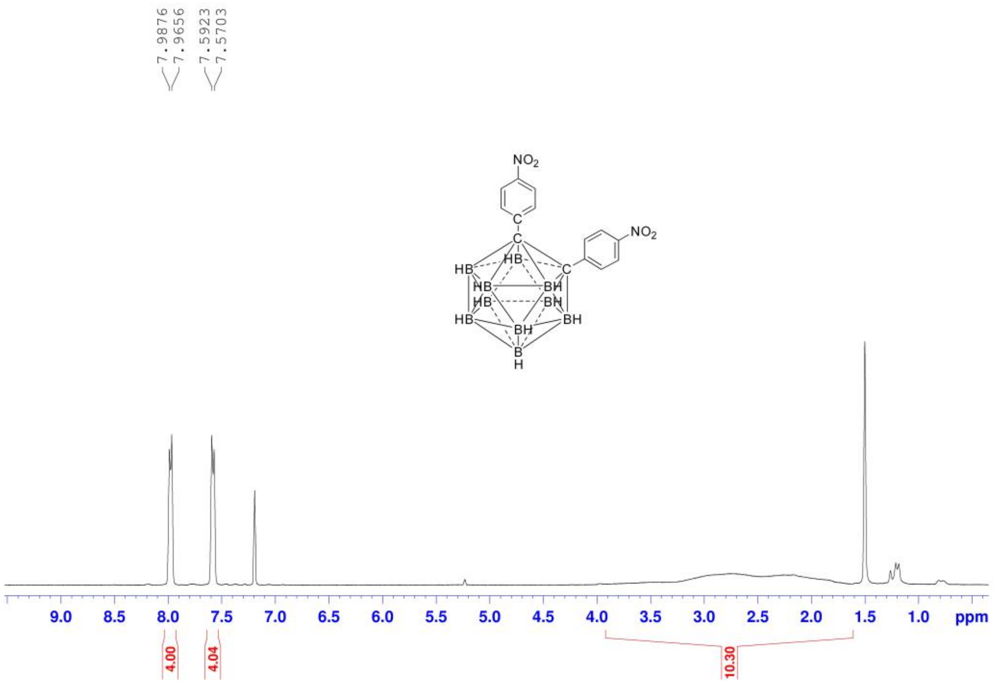

Supplementary Figure 5. ^{1}H NMR spectrum of compound CB-NO_2 ($CDCl_3$/TMS, 400 MHz, ppm).

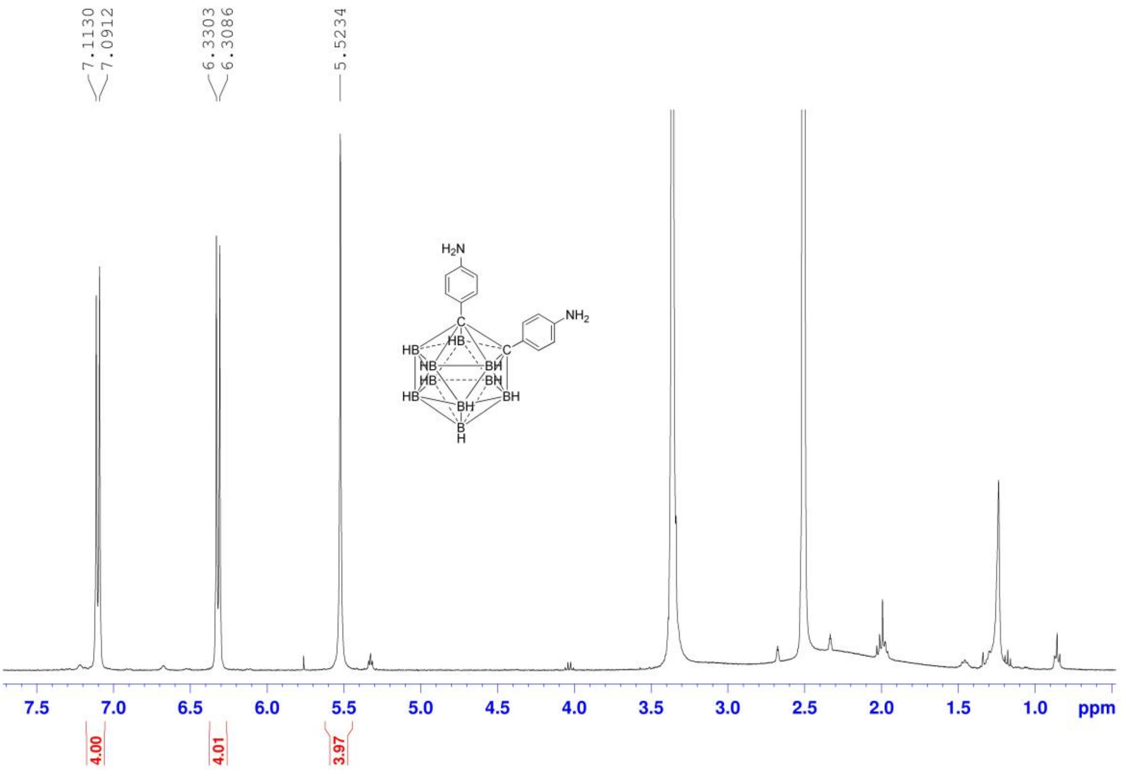

Supplementary Figure 6. ^{1}H NMR spectrum of compound CB-NH_2 (DMSO-d_6/TMS, 400 MHz, ppm).

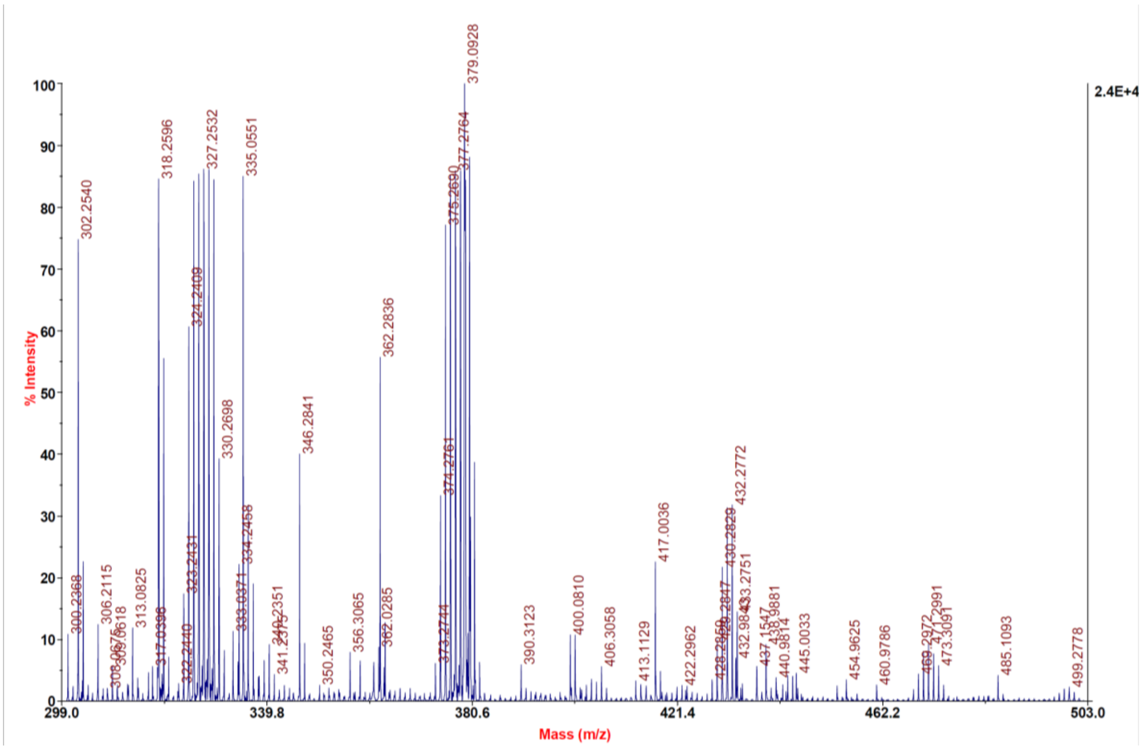

Supplementary Figure 7. Mass spectra of CB-NH_2.

Supplementary Note 1

Synthetic details:

1,2-bis(4-phenyl)-1,2-dicarba-closo-decaborane (**CB-ph**)

dodecahydro-arachno-bis(acatonitrile)decaborane (1021 mg, 5 mmol), diphenylacetylene (979 mg, 5.5 mmol), and silver nitrate (84.9 mg, 0.5 mmol) were added to a 100 mL three-neck flask. The reactant was degassed and flushed with dry nitrogen for several times. Then dry toluene (50 mL) was added to dissolved the reactant. The mixture was heated to 80 °C with stirring for 12 h, cooled down and quenched with methanol (10 mL). The mixture was then extracted with CH_2Cl_2, washed with brine, and dried over anhydrous Na_2SO_4. At last, the solvent was removed by rotary evaporation, and crude product was purified by column chromatography silica gel (petroleum ether) to give a white solid in 42% yield (626 mg).

^{1}H NMR ($CDCl_3$/TMS, 400 MHz, δ, ppm):7.41-7.43(d, J=7.5 Hz, 4H, phenyl-H), 7.24(m, 2H, phenyl-H), 7.15(m, 4H, phenyl-H), 1.6-4.0(br, m, 10H, boron-H).

1,2-bis(4-nitrobenzene)-1,2-dicarba-closo-dodecaborane (**CB-NO$_2$**)

A mixture of H_2SO_4 (97% w/w, 32 mL) and HNO_3 (61% w/w, 6.4 mL) was stirred and cooled in ice-bath. 1,2-dicarba-closo-decaborane (1193.6 mg, 4 mmol) was dissolved in 40 mL CH_2Cl_2 and added dropwise. Then the solution was stirred at room temperature for 6 h. The mixture was poured into ice-cold water (100 mL) and extracted with CH_2Cl_2. The organic phase was washed with saturated $NaHCO_3$ solution and brine, then dried over anhydrous Na_2SO_4. The solvent was removed by rotary evaporation, and crude product was purified by column chromatography silica gel (petroleum ether: EtOAc, 8:1) to give a pale yellow solid (233 mg, 15%).

^{1}H NMR ($CDCl_3$/TMS, 400 MHz, δ, ppm):7.96-7.98(d, J=8.8 Hz, 4H, phenyl-H), 7.57-7.59(d, J=8.8 Hz, 4H, phenyl-H), 1.6-4.0(br, m, 10H, boron-H).

1,2-bis(4-aminophenyl)-1,2-dicarba-closo-dodecaborane (**CB-NH$_2$**):

1,2-bis(4-nitrobenzene)-1,2-dicarba-closo-dodecaborane (100 mg, 0.26 mmol), $SnCl_2 \cdot 2H_2O$ (593 mg, 2.63 mmol), ethanol, and hydrochloric acid (36%, w/w, 1.5 mL) were added into a 250 mL three-neck flask. The reactant was degassed and flushed with dry nitrogen for several times, then stirring at 90 °C for 16 h. The mixture was cooled down, and triphenylamine (6 mL) was added to adjust the PH to around 8. Then the mixture was extracted with CH_2Cl_2 and the organic phase was washed with brine, then dried over anhydrous Na_2SO_4. The solvent was removed by rotary evaporation, and crude product was purified by column chromatography silica gel (petroleum ether: EtOAc, 2:1) to give a pale yellow solid in 85.0 % yield (71.85 mg, 0.22 mmol).

^{1}H NMR (DMSO-d_6/TMS, 400 MHz, δ, ppm), 7.11(d, J=8.7 Hz, 4H, phenyl-H), 6.33(d, J=8.7 Hz, 4H, phenyl-H), 5.52(br, s, 4H, amiono-H), 1.5-3.0(br, m, 10H, boron-H). Mass spectrometry (MALDI-TOF-MS: m/z); Calculated for $C_{14}H_{21}B_{10}N_2$: 327.26; found: 327.25.

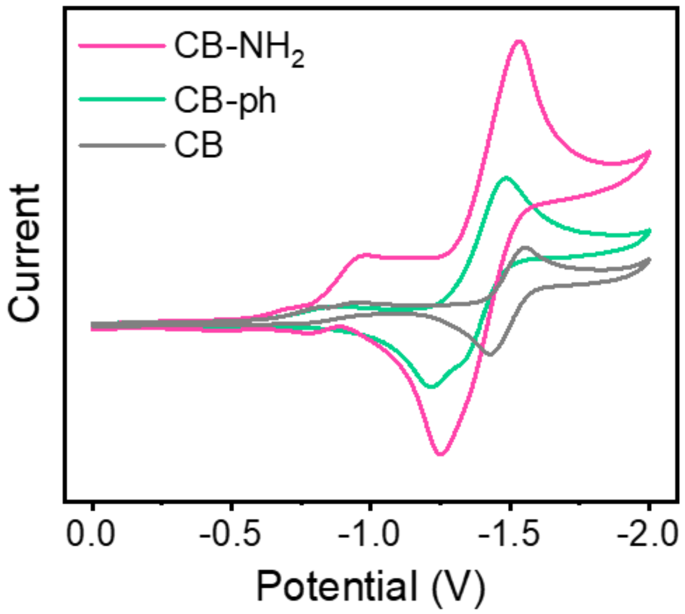

Supplementary Figure 8. The LUMO energy level is estimated from the first formal reduction peak in cyclic voltammetry (CV) curves with using ferrocene/ferrocenium (Fc/Fc^+) as reference. Subsequently, the HOMO energy level is obtained by subtracting LUMO from the energy gap.

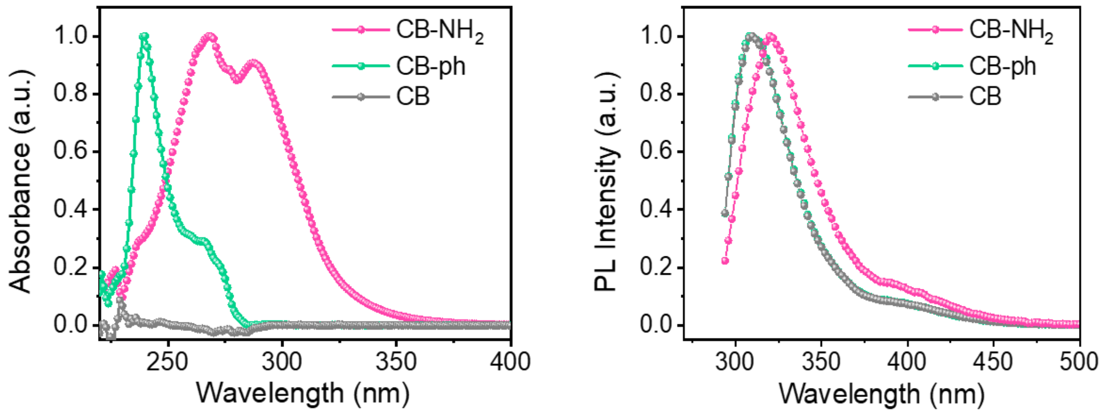

Supplementary Figure 9. UV-vis absorption and emission spectra (λ_{exc} = 235 nm) of in THF (1×10^{-5} M).

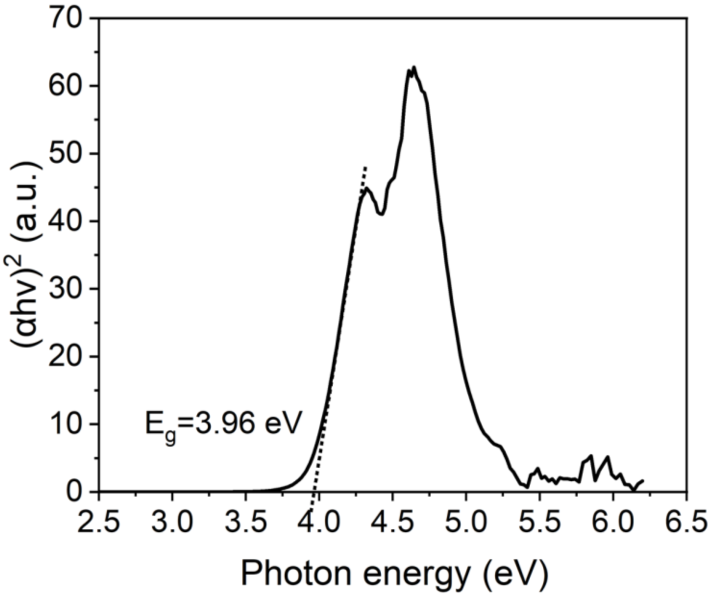

Supplementary Figure 10. Tauc plots of the absorption spectra of CB-NH_2, the optical bandgap is estimated to 3.96 eV.

Supplementary Table 1. Energy levels based on cyclic voltammetry and UV-vis absorption and emission spectra.

	E_{HOMO}	λ_{onset}	Eg	E_{LUMO}
CB-NH_2	-7.37	304.26	4.07	-3.30
CB-ph	-7.73	281.84	4.40	-3.33
CB				-3.91

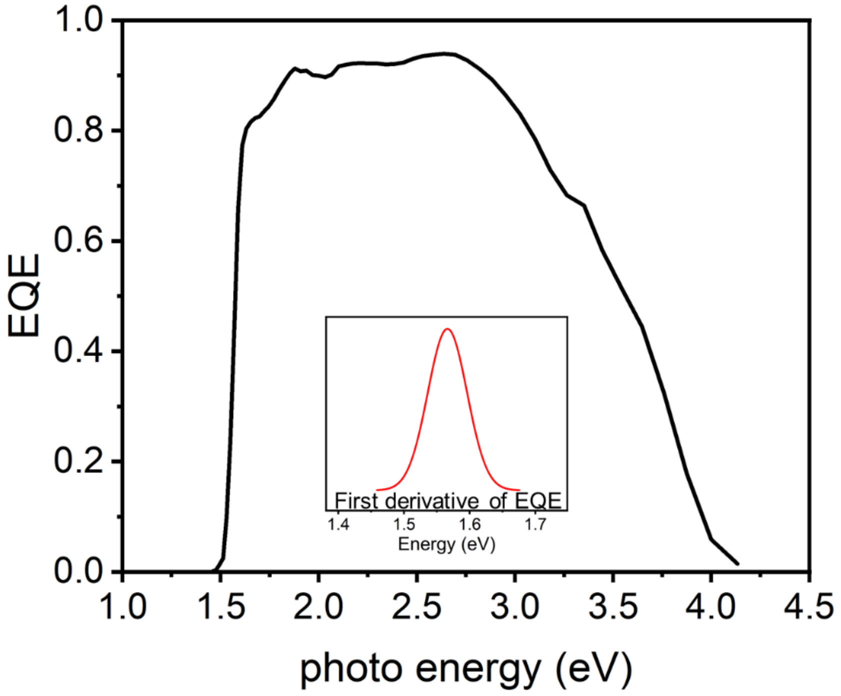

Supplementary Figure 11. External quantum efficiency (EQE) and d(EQE)/dE versus photon energy in the inset confirming a bandgap of 1.56 eV.

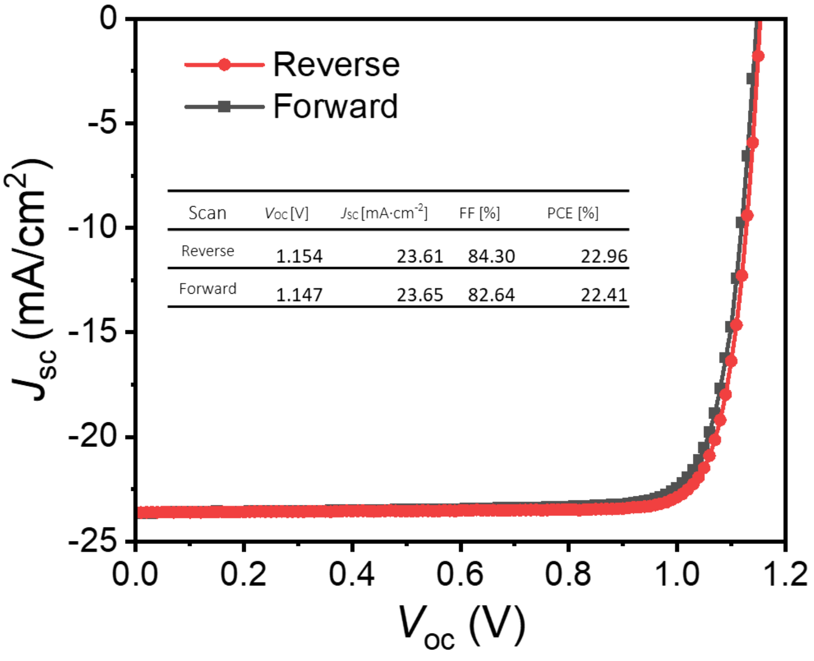

Supplementary Figure 12. Hysteresis of typical CB-NH_2 based device at a scan speed of 20 mV/s.

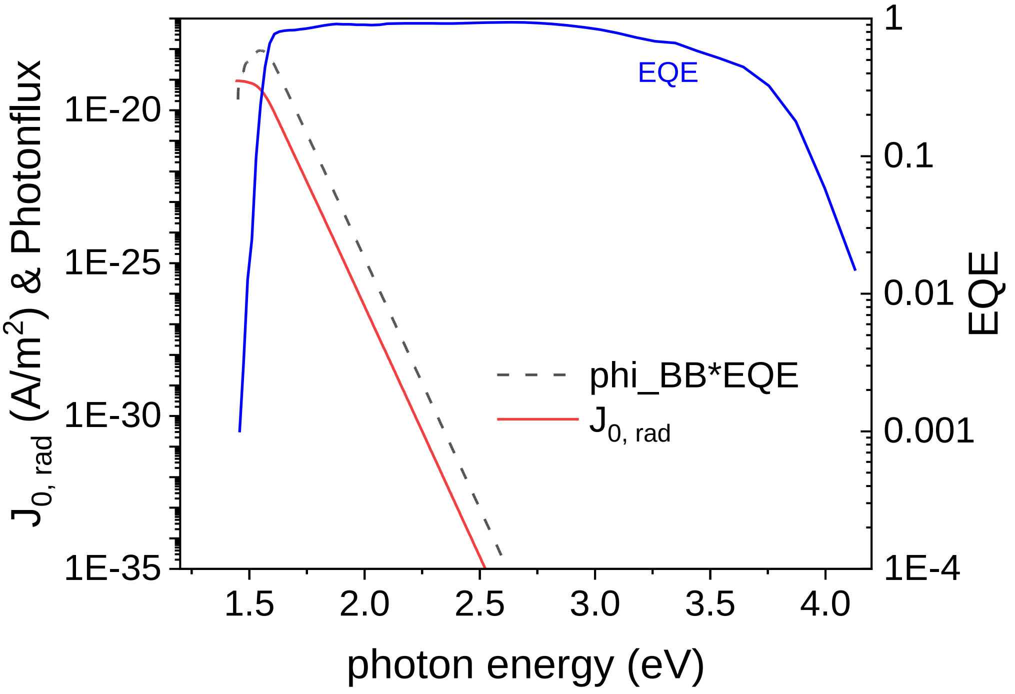

Supplementary Figure 13. External quantum efficiency (EQE) of the *pin* PSCs and the emitted spectral photon flux calculated when the device is in equilibrium with the black-body (BB) radiation of the environment at 300 K, the $J_{0,rad}$ is calculated to $9x10^{-20}$ A/m^2.

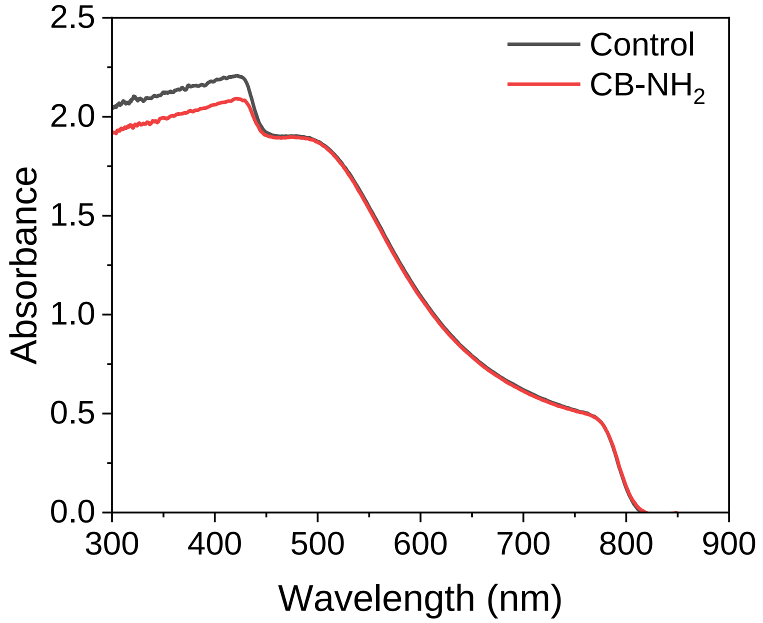

Supplementary Figure 14. Absorption spectra of control and CB-NH_2 treated films.

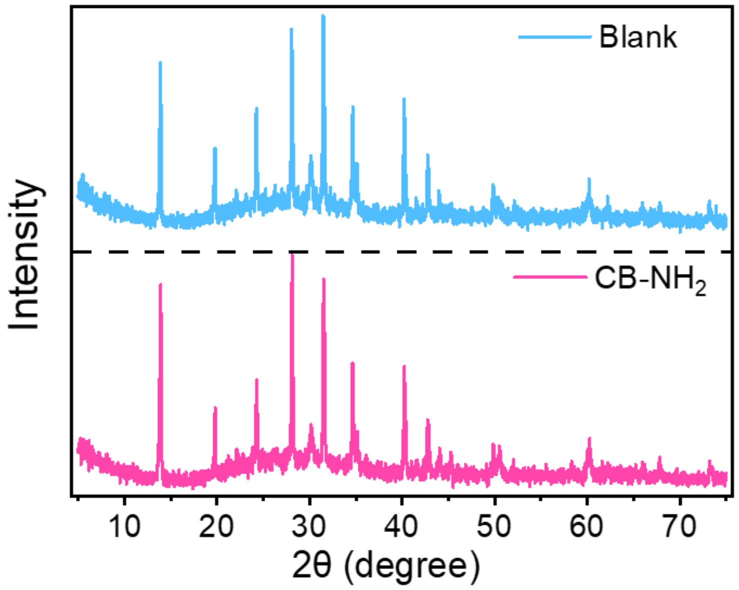

Supplementary Figure 15. XRD spectra of perovskite film and $CB\text{-}NH_2$ coated perovskite film, both of which have been annealed at 100°C for 10 mins.

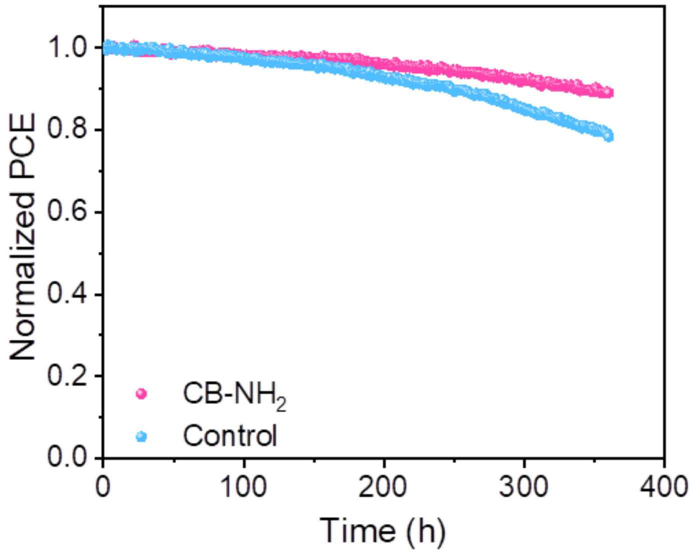

Supplementary Figure 16. Maximum power point tracking under 1 sun illumination of $CB\text{-}NH_2$ based and control PSCs in air (~30% RH and ~40 °C).

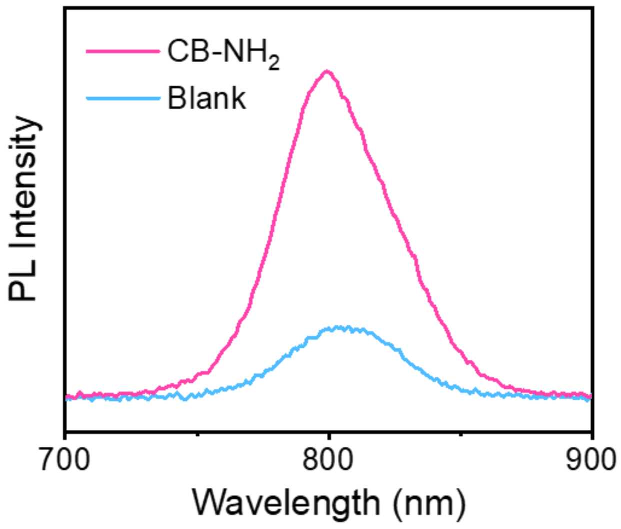

Supplementary Figure 17. Steady state PL spectrum of bare perovskite film and CB-NH_2 coated perovskite film.

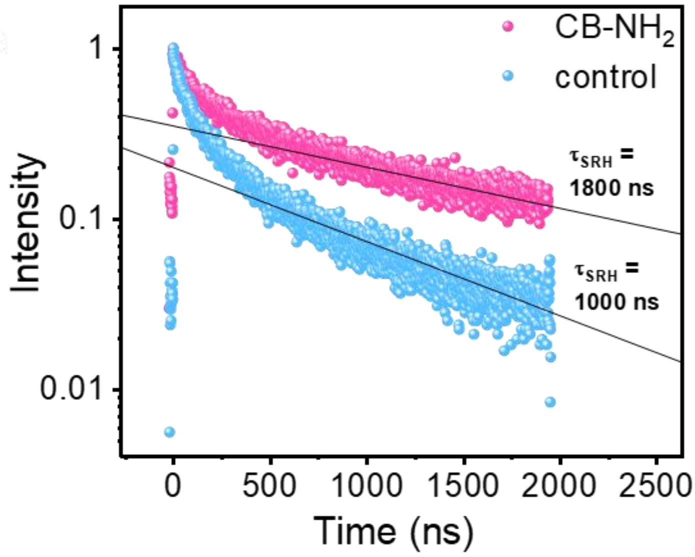

Supplementary Figure 18. TRPL monoexponential (Shockley-Read-Hall) lifetimes of bare perovskite film and CB-NH_2 coated perovskite film fabricated on quartz measured at an excitation laser wavelength of 520 nm.

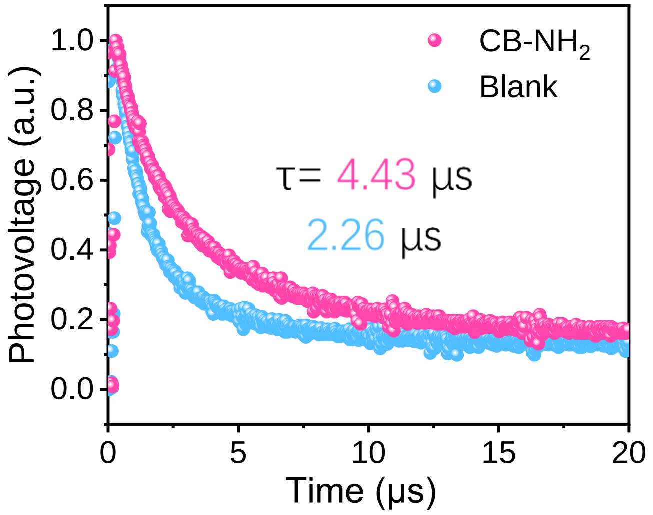

Supplementary Figure 19. Transient photovoltage test on complete PSCs with and without CB-NH_2 interlayer between perovskite and C_{60}, showing that CB-NH_2 improves carrier lifetime. The intensity of the background white light (LED) was adjusted to produce near V_{OC} (1.1 V) on the devices to simulate 1 sun condition.

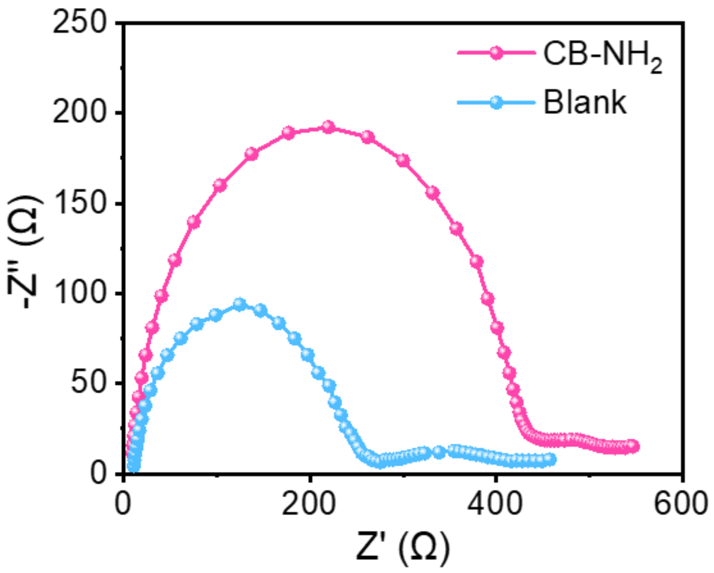

Supplementary Figure 20. Impedance spectrum of PSCs with and without CB-NH_2 interlayer. The spectrum was performed using a two-electrode system under dark with electrochemical workstation (Zahner IM6e).

Electron only devices

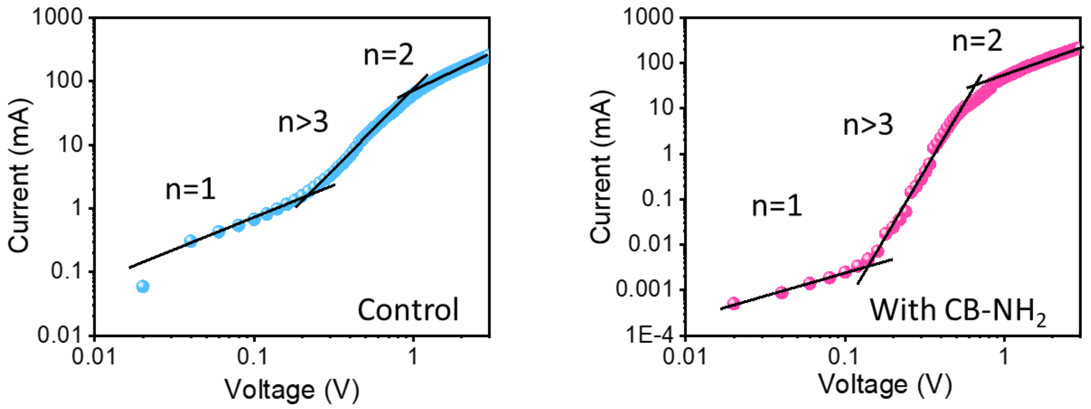

Supplementary Figure 21. Dark *JV* profiles of the electron-only devices with a structure of ITO/SnO_2/perovskite (with or without CB-NH_2)/PCBM/Au.

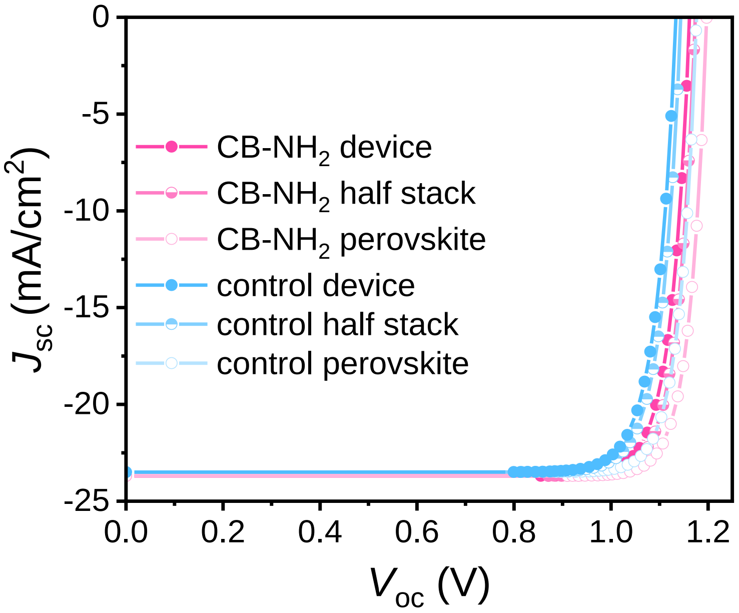

Supplementary Figure 22. Pseudo *JV* curves of neat perovskite film, perovskite/C_{60} half stack, and full device, comparing the influence of CB-NH_2 treatment.

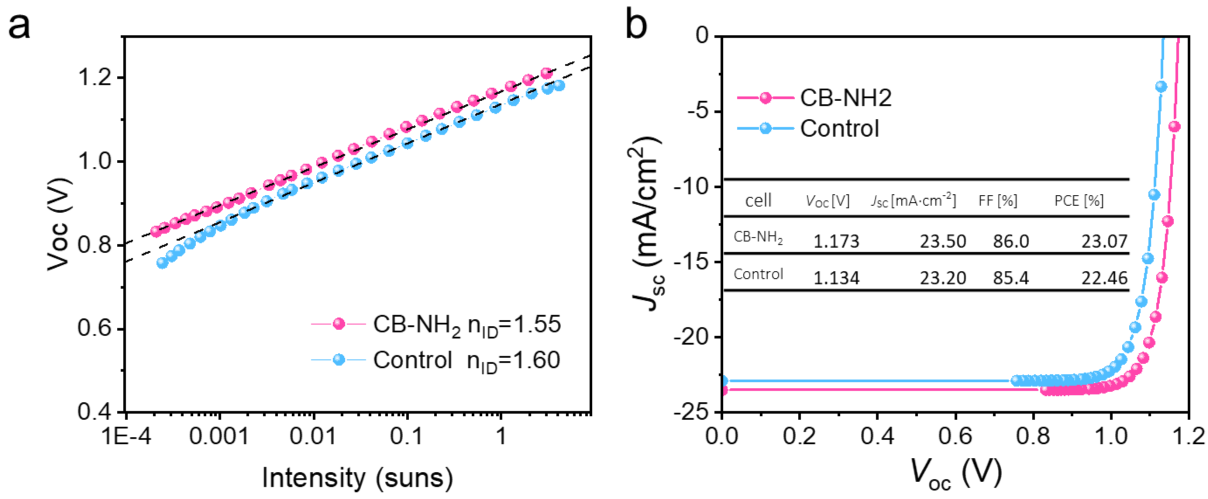

Supplementary Figure 23. a Intensity dependent V_{OC} measurements of the devices based on w/wo CB-NH_2 samples. The ideality factor (n_{ID}) values are shown in the graph. **b** Pseudo *JV* curves from V_{OC} - intensity spectrum based on w/wo CB-NH_2 samples.

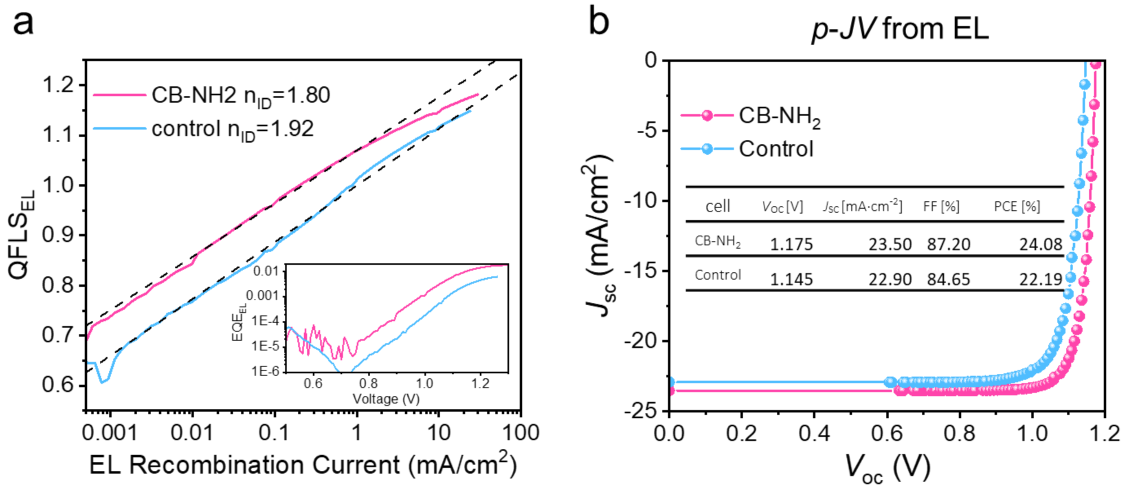

Supplementary Figure 24. a Intensity dependent QFLS as obtained from EL measurement, the inset is the EQE_{EL} versus applied voltage. **b** Pseudo *JV* curves from $QFLS_{EL}$ - intensity spectrum based on w/wo CB-NH_2 samples.

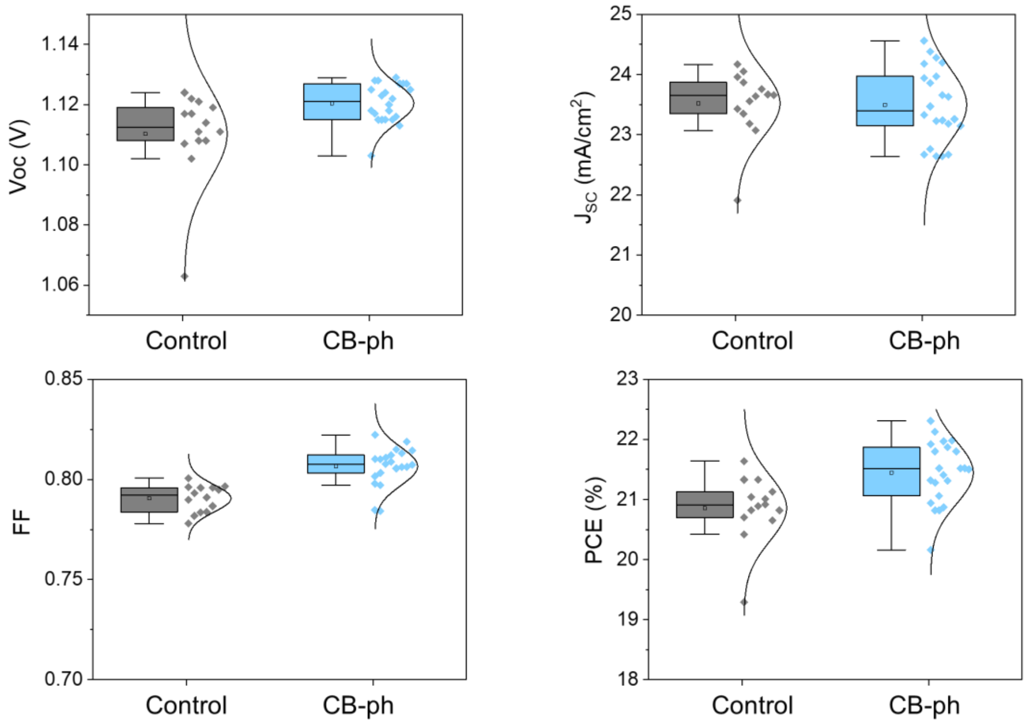

Supplementary Figure 25. Parameters distribution of V_{OC}, J_{SC}, FF, and PCE for control, and CB-ph treated devices, respectively.

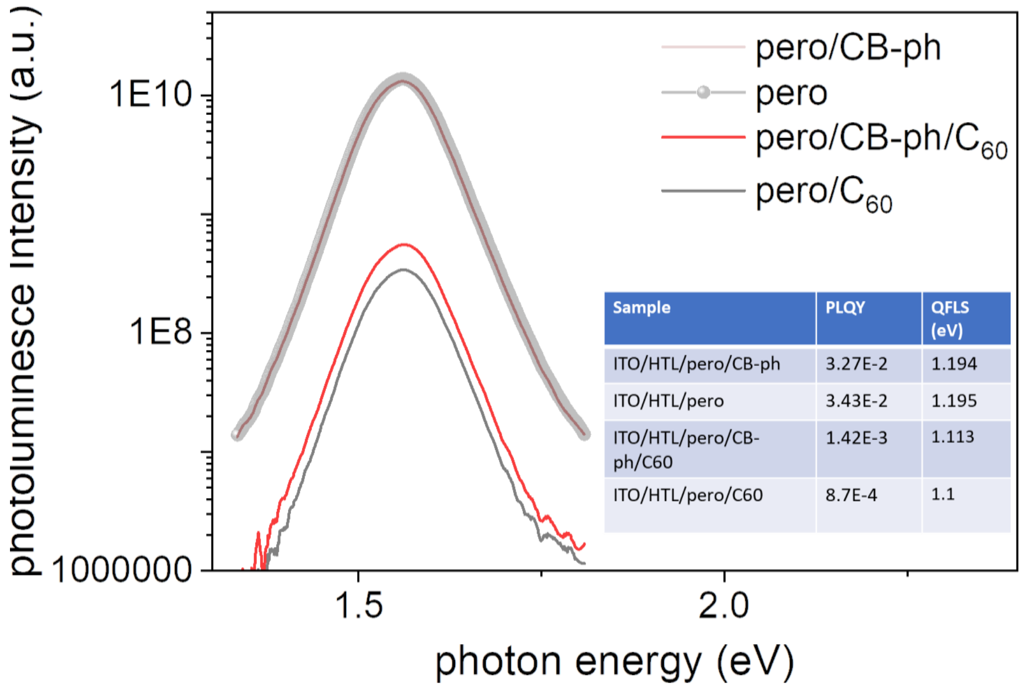

Sample	PLQY	QFLS (eV)
ITO/HTL/pero/CB-ph	3.27E-2	1.194
ITO/HTL/pero	3.43E-2	1.195
ITO/HTL/pero/CB-ph/C60	1.42E-3	1.113
ITO/HTL/pero/C60	8.7E-4	1.1

Supplementary Figure 26. Photoluminescence spectrum and the calculated photoluminescence quantum yield measurements (PLQY) and internal quasi-Fermi level splitting (QFLS) with/without CB-ph.

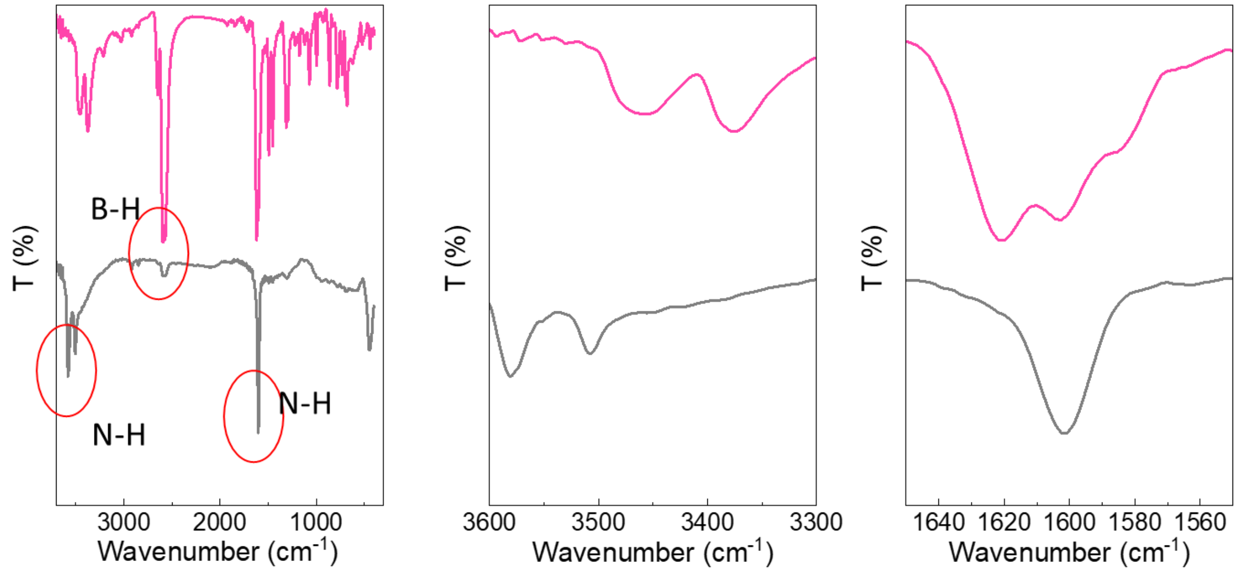

Supplementary Figure 27. FTIR spectrum of pure CB-NH_2 (grey) and CB-NH_2 mixed with PbI_2 (pink).

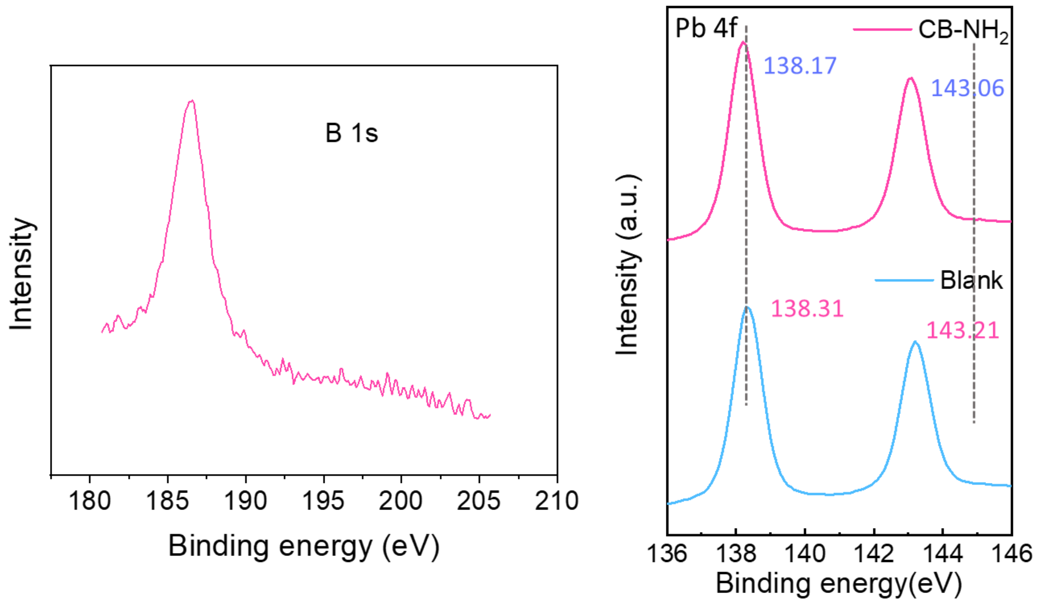

Supplementary Figure 28. XPS spectra of the Pb 4f and B 1s core levels for the pristine perovskite film and the CB-NH_2 coated film.

Supplementary Note 2

DFT calculation details. First principles density functional theory (DFT) calculations were carried out with the Perdew–Burke–Ernzerhof (PBE) functional using the Vienna ab initio simulation package (VASP)[2–4]. To correctly describe the geometric and electronic structure of $FAPbI_3$, a DFT+U (I:8 eV, Pb: 9 eV) method[5,6], including the Van der Waals interaction (DFT-D3) and spin-orbit coupling (SOC) effect, was employed[7–11]. For the cubic $FAPbI_3$, a large (2√2×2√2) stoichiometric PbI-terminated (001) surface corresponding to a size of 18.13×18.13×27.92 $Å^3$ with three atom-unit layers was constructed to investigate the PbI-rich perovskite surface. The vacuum layer is 15 Å. In the structural optimization, the bottom one layer was fixed whereas the top two layers were fully relaxed. The energy cutoff of the plane wave basis set is 400 eV. The Brillouin zone was sampled using the Monkhorst-Pack scheme with a 1×1×1 k-point mesh in the geometry optimization owing to the large slab size. All the optimized structures were obtained by minimizing the forces until they fell below 0.05 eV/Å for each atom. For the introduction of iodine Frenkel defects on the $FAPbI_3$(001) surface, we constructed and calculated as many as 42 possible configurations to obtain the energetically most favorable one.

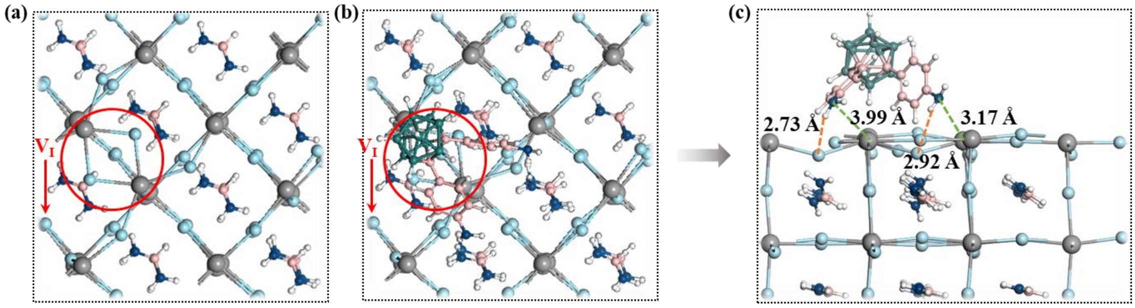

Supplementary Figure 29. Top views of PbI_2-terminated surfaces with iodine Frenkel defect, respectively. (a) without and (b) without CB-NH2 adsorbed, respectively. (c) are the relative side views of (b) structures. The iodine Frenkel defect formation energy increases from 0.22 eV to 0.39 eV. Notes: the I-vacancy (V_I), the iodine Frenkel pair, and the length of N-H···I/N-Pb are depicted. Color scheme: Pb: grey; I: light bule; C: pink; N: dark blue; B: green; H: white.

Supplementary References:

1. Kavadiya, S. *et al.* Investigation of the Selectivity of Carrier Transport Layers in Wide-Bandgap Perovskite Solar Cells. *Solar RRL* **5**, 2100107 (2021).
2. Perdew, J. P., Burke, K. & Ernzerhof, M. Generalized Gradient Approximation Made Simple. *Phys. Rev. Lett.* **77**, 3865–3868 (1996).
3. Kresse, G. & Furthmüller, J. Efficiency of ab-initio total energy calculations for metals and semiconductors using a plane-wave basis set. *Comput. Mater. Sci.* **6**, 15–50 (1996).
4. Kresse, G. & Hafner, J. Ab initio molecular-dynamics simulation of the liquid-metal--amorphous-semiconductor transition in germanium. *Phys. Rev. B* **49**, 14251–14269 (1994).
5. Peng, C., Chen, J., Wang, H. & Hu, P. First-Principles Insight into the Degradation Mechanism of $CH_3NH_3PbI_3$ Perovskite: Light-Induced Defect Formation and Water Dissociation. *J. Phys. Chem. C* **122**, 27340–27349 (2018).
6. Peng, C., Wang, J., Wang, H. & Hu, P. Unique Trapped Dimer State of the Photogenerated Hole in Hybrid Orthorhombic $CH_3NH_3PbI_3$ Perovskite: Identification, Origin, and Implications. *Nano Lett.* **17**, 7724–7730 (2017).
7. Grimme, S., Ehrlich, S. & Goerigk, L. Effect of the damping function in dispersion corrected density functional theory. *J. Comput. Chem.* **32**, 1456–1465 (2011).
8. Even, J., Pedesseau, L., Jancu, J.-M. & Katan, C. Importance of Spin–Orbit Coupling in Hybrid Organic/Inorganic Perovskites for Photovoltaic Applications. *J. Phys. Chem. Lett.* **4**, 2999–3005 (2013).
9. Amat, A. *et al.* Cation-Induced Band-Gap Tuning in Organohalide Perovskites: Interplay of Spin–Orbit Coupling and Octahedra Tilting. *Nano Lett.* **14**, 3608–3616 (2014).
10. Menéndez-Proupin, E., Palacios, P., Wahnón, P. & Conesa, J. C. Self-consistent relativistic

band structure of the $CH_3NH_3PbI_3$ perovskite. *Phys. Rev. B* **90**, 045207 (2014).

11. Umari, P., Mosconi, E. & De Angelis, F. Relativistic GW calculations on $CH_3NH_3PbI_3$ and $CH_3NH_3SnI_3$ Perovskites for Solar Cell Applications. *Sci Rep* **4**, 4467 (2014).
