# Supplementary material for: Overcoming C60-induced interfacial recombination in inverted perovskite solar cells by electron-transporting carborane
Source: Nat Commun. 2022 Dec 2;13:7454. doi: 10.1038/s41467-022-34203-x (PMC9718752; doi:10.1038/s41467-022-34203-x)
Supplement: Supplementary file 3 — Solar Cells Reporting Summary [file 41467_2022_34203_MOESM3_ESM.pdf]

nature research

Corresponding author(s): Martin Stolterfoht and Yongzhen Wu

Solar Cells Reporting Summary

Nature Research wishes to improve the reproducibility of the work that we publish. This form is intended for publication with all accepted papers reporting the characterization of photovoltaic devices and provides structure for consistency and transparency in reporting. Some list items might not apply to an individual manuscript, but all fields must be completed for clarity.

For further information on Nature Research policies, including our data availability policy, see Authors & Referees.

Experimental design

Please check: are the following details reported in the manuscript?

1. Dimensions

Item	Yes/No	Details
Area of the tested solar cells	☒ Yes ☐ No	Page 2 in SI: "The active area of tested cells was 12 mm^2".
Method used to determine the device area	☒ Yes ☐ No	Page 2 in SI: "as defined from the area of overlap of the electrodes."

2. Current-voltage characterization

Item	Yes/No	Details
Current density-voltage (J-V) plots in both forward and backward direction	☒ Yes ☐ No	Reported in Supplementary Fig. S8.
Voltage scan conditions<br>*For instance: scan direction, speed, dwell times*	☒ Yes ☐ No	Page 2 in SI: "The temperature of the cell was fixed to 25 °C and a voltage ramp of 20 mV/s was used. "
Test environment<br>*For instance: characterization temperature, in air or in glove box*	☒ Yes ☐ No	Page 2 in SI: "The temperature of the cell was fixed to 25 °C and a voltage ramp of 20 mV/s was used. "
Protocol for preconditioning of the device before its characterization	☐ Yes ☒ No	No pre-conditioning protocol was used.
Stability of the J-V characteristic<br>*Verified with time evolution of the maximum power point or with the photocurrent at maximum power point; see ref. 7 for details.*	☒ Yes ☐ No	Reported in Figure 2e (stabilized current at fixed voltage) and Figure 3c (evolution of MPP).

3. Hysteresis or any other unusual behaviour

Item	Yes/No	Details
Description of the unusual behaviour observed during the characterization	☒ Yes ☐ No	We typically observe a dip in the PCE in the initial 10 seconds which we attribute to current losses related to ion movement in the perovskite absorber layer, ref. 44,45.
Related experimental data	☒ Yes ☐ No	Figure 2e and the corresponding description in the manuscript.

4. Efficiency

Item	Yes/No	Details
External quantum efficiency (EQE) or incident photons to current efficiency (IPCE)	☒ Yes ☐ No	Figure 2f in the manuscript.
A comparison between the integrated response under the standard reference spectrum and the response measure under the simulator	☒ Yes ☐ No	Figure 2f, the integrated product of EQE and solar cell spectrum matches the current under the sun simulator within 2%.
For tandem solar cells, the bias illumination and bias voltage used for each subcell	☐ Yes ☒ No	Not applicable here .

5. Calibration

Item	Yes/No	Details
Light source and reference cell or sensor used for the characterization	☒ Yes ☐ No	An Oriel class AAA Xenon lamp-based sun simulator was used for illumination. The intensity of the solar simulator was monitored during the JV scans using a Si diode.
Confirmation that the reference cell was calibrated and certified	☒ Yes ☐ No	The intensity was calibrated with a KG5-filtered silicon solar cell calibrated by Fraunhofer ISE.

Calculation of spectral mismatch between the reference cell and the devices under test	☒ Yes ☐ No	The spectral mismatch factor (S_M) was calculated to be 0.982 for the optimized devices with EQE spectra presented in Figure 1f, however, the obtained current has not been upscaled by 1/S_M.

6. Mask/aperture

Size of the mask/aperture used during testing	☒ Yes ☐ No	Page 2 in SI: "An illumination mask with an area of 9 mm2 was used to measure the JV-characteristics."
Variation of the measured short-circuit current density with the mask/aperture area	☒ Yes ☐ No	JV results in main text are reported with illumination mask. We note, the unmasked current is typically ~5% higher.

7. Performance certification

Identity of the independent certification laboratory that confirmed the photovoltaic performance	☐ Yes ☒ No	No certification was carried out since no new efficiency record is reported.
A copy of any certificate(s)<br>*Provide in Supplementary Information*	☐ Yes ☒ No	Not applicable.

8. Statistics

Number of solar cells tested	☒ Yes ☐ No	Figure 2a-c in the manuscript.
Statistical analysis of the device performance	☒ Yes ☐ No	In caption in Figure 2 in the manuscript.

9. Long-term stability analysis

Type of analysis, bias conditions and environmental conditions<br>*For instance: illumination type, temperature, atmosphere humidity, encapsulation method, preconditioning temperature*	☒ Yes ☐ No	Long term stability tests were carried out as shown in Figure 3c (evolution of MPP at 26 °C, inert atmosphere in glovebox, white light LED) and repeated in Supplementary Figure S12 (evolution of MPP at 40 °C, ~30% RH outside, unencapsulated, white light LED).
